# The role of Empathy in the relationship between emotional support and caring behavior towards patients among intern nursing students

**DOI:** 10.1186/s12912-024-02074-w

**Published:** 2024-06-28

**Authors:** Mohamed Hussein Ramadan Atta, Heba Abdel-Hamid Hammad, Nadia Waheed Elzohairy

**Affiliations:** 1 Psychiatric and mental health Nursing, Psychiatric and mental- health nursing Department, Faculty of Nursing, Alexandria University, 9 Edmond Vermont Street, Smouha, Alexandria City, Egypt; 2 Faculty of Nursing, Psychiatric Nursing Department, Admeon Freemon ST, Semoha, Alexandria City, Egypt; 3 Psychiatric Nursing and Mental Health, Faculty of Nursing, Damnhour University, Damnhour City, Egypt

**Keywords:** Caring Behavior, Emotional support, Empathy, Intern nursing students, Mediating role, Nurse-patient relationship

## Abstract

**Background:**

The empathic relationship between nursing students and patients allows them to understand and address caring behavior for patients. Appropriate emotional support equips them to overcome the complexities and difficulties inherent in patient care. This support cultivates resilience and self-awareness, enabling students to manage their emotions effectively and establish meaningful connections and caring with their patients.

**Objectives:**

To investigate the role of empathy in the association between emotional support and caring behavior toward patients among intern nursing students at Alexandria and Damanhur University.

**Subjects:**

The study subjects were 200 intern nursing students in their internship years of 2022–2023, randomly selected from an equal sample size from Alexandria and Damanhur University, Egypt.

**Tools:**

A questionnaire of social information& academics from students, the Toronto Empathy Questionnaire, the Caring Dimension Inventory, and the Multidimensional Scale of Perceived Social Support are used to collect participants’ data.

**Results:**

Empathy was associated with caring behavior and emotional support among nursing students (*P* < 0.001), and higher levels of empathy indicated increased levels of caring behavior and emotional support. The caring behavior significantly increased when intern nursing students received more emotional support and among those who were not working in private hospitals (*p* < 0.001,&*p* = 0.023 respectively). Empathy acts as a mediating role in the relationship between emotional support and caring behavior.

**Implications:**

Implementing strategies to assist interns in navigating challenges and promoting a culture of support can facilitate the cultivation of caring behaviors. Shedding light on the interconnectedness of empathy, emotional support, and caring behavior can inform the design of interventions to strengthen empathy as a pathway to improving patient outcomes.

**Conclusion:**

Empathy is a mediating factor in the relationship between emotional support and caring behavior. This suggests that interventions promoting empathy may serve as a pathway to enhancing caring behavior among nursing students and strategies for improving patient care outcomes by strengthening empathy skills among healthcare professionals.

## Introduction

Internship experience offers nursing students a valuable opportunity to enhance their professional development by gaining practical experience in a field that aligns with their academic pursuits or desired careers. In nursing, intern students play a significant role in delivering high-quality patient care [1, 2]. In Egypt, intern nursing students are vital to the healthcare system as they provide patient care under the supervision of experienced nurses and clinical instructors. Their responsibilities include indispensable assisting with patient assessment, administering medications, monitoring vital signs, and providing essential care such as bathing and feeding. Additionally, nursing interns are crucial in patient education, offering information on health promotion and disease prevention [3, 4].

Furthermore, intern nursing students in Egypt are essential members of the healthcare workforce, especially in University hospitals. They also have an invaluable impact on patient outcomes. As new entrants in the care field, intern nursing students should possess empathy and demonstrate caring behavior towards their patients. These factors greatly influence patients’ well-being and health outcomes [5, 6]. Moreover, intern nursing students will be able to gradually improve their caring behavior and empathy toward patients by receiving emotional support from others [1, 7]. Intern nursing students who receive emotional support and exhibit empathy are more likely to engage in caring behaviors. This enhances care quality, patient satisfaction, and improved health outcomes [8, 9]. Nursing students can receive emotional support within a social context, be it from family, friends, or significant others, and form a crucial web of connections that sustains and nurtures individual well-being. Each relationship contributes a unique blend of understanding, empathy, and encouragement, creating a tapestry of support that strengthens the fabric of one’s emotional resilience. Significant others as clinical educators, their role extends beyond imparting clinical knowledge; they become trusted allies who understand the unique challenges of nursing education and offer empathetic support, fostering a sense of belonging and confidence in the student’s ability to navigate the complexities of their chosen profession [10, 11].

Empathy stands out as an essential trait and a valuable asset to be possessed by graduate nurses. It is identified as the ability to comprehend and share the feelings of others. According to the American Nurses Association (2021), empathy is entering into, being sensitive to, and understanding another person’s feelings, thoughts, and experiences without having those feelings, thoughts, and experiences of oneself [12–15]. An empathic nurse can help establish a strong rapport with her patient, understand his unique needs and circumstances, and provide compassionate care. Empathizing with the patients can provide more personalized care and support, leading to better patient health outcomes and improved patient satisfaction [1, 8, 13].

In addition, empathy can also help intern nursing students manage their own emotions and cope with the stress and challenges of their profession, which in turn helps them provide emotional support to their patients. Empathy is critical for nurses to enhance the quality of care and strengthen the nurse-patient relationship [16, 17]. In this respect, Korkmaz Doğdu et al. (2022) conducted a correlational study on nursing students’ empathy levels and caring behavior perceptions. This research reveals the significant influence of a student’s development on their empathy and caring behaviors. As students progress in their educational levels, there is a corresponding increase in both empathy and caring behaviors. It has been established that maintaining empathetic understanding and possessing practical communication skills positively contribute to caring behaviors. This study distinctly emphasizes the crucial role of nurturing ontological caring competencies in the development of nursing students [18].

Emotional support is of utmost importance for the well-being of nurses, particularly intern students, who often face high levels of stress and emotional exhaustion due to the demands of their professional careers. According to Kort-Butler (2017), emotional support perception is a term rooted in the broader concept of social support, encompassing receiving care, consideration, empathy, affection, and trust (19). Emotional support is crucial to address these challenges and promote mental health and job satisfaction. Recognizing and offering emotional support to nurses is essential, as it can significantly enhance their job performance and overall well-being. Creating a therapeutic environment through emotional support facilitates self-healing and promotes well-being. Intern nursing students may receive support from their families, colleagues, and academic supervisors. Previous research demonstrated that emotional support from colleagues and supervisors is a crucial predictor of new nurses’ job satisfaction, intention to remain in their profession, and ability to deliver compassionate care to their patients [11, 19–21].

A study by Zhou et al., 2022 found an association between perceived emotional support and psychological well-being among nursing students. This is clarified through the mediating factors of self-compassion and professional self-concept. A positive association between heightened self-compassion and a more favorable professional self-concept is noted. This suggests that perceived emotional support can foster self-compassion and a positive professional self-concept, enhancing psychological well-being. These findings emphasize the need for emotional support to improve nursing students’ assets and improve nursing care [22].

Caring behavior is a fundamental aspect of nursing care, including actions and attitudes that demonstrate compassion, empathy, and respect toward patients and their families. The International Council of Nurses (2012) defines caring as the “essence of nursing,” emphasizing establishing therapeutic relationships that acknowledge individual patient needs and promote well-being. Caring behaviors manifest in various forms, including providing emotional support, administering medications, and advocating for patients’ rights and preferences [9, 23, 24].

Furthermore, caring behavior plays a critical role in patient-centered care. It prioritizes involving patients and their families in the care process and delivering personalized care tailored to their needs and circumstances. When intern nursing students exhibit caring behaviors, they enhance the quality of care and foster a nurturing environment that supports patients’ recovery and well-being. In summary, caring behavior improves patient outcomes and is vital to nursing care. The nurse’s perception of emotional support and empathy can influence patient caring behavior [23–26].

Despite the existing knowledge on empathy, emotional support, and caring behavior, a paucity of research explicitly articulates the intricate interplay between these variables, especially in the context of intern nursing students. Few studies may have explored empathy as a mediator between emotional support and caring behavior. Yet, the unique challenges and dynamics faced by intern nursing students during their training were not addressed [16, 19, 26–28].

In the context of intern nursing students, it is hypothesized that the emotional support they receive may influence their empathy towards patients, affecting their caring behavior. When nursing students receive emotional support from their educators, mentors, or peers, they may develop a greater capacity for empathy toward patients. Through guidance, encouragement, and constructive feedback, clinical educators and staff help alleviate the stress and anxiety often associated with rigorous training. This enhanced empathy can translate into more caring and compassionate behaviors when interacting with patients. Understanding the role of empathy in this relationship is crucial for nursing education and practice. By recognizing the impact of emotional support on empathy and subsequent caring behavior, educators and mentors can emphasize the importance of creating a supportive learning environment that nurtures empathy development in nursing students. This, in turn, can contribute to cultivating a caring and empathetic healthcare workforce that prioritizes patient well-being and satisfaction [1, 2, 28]. Therefore, this study aims to identify the role of empathy in the relationship between the perception of emotional support and caring behavior toward patients among intern nursing students.

### The study objectives are to

- Investigate how empathy plays a role in shaping the relationship between emotional support and caring behavior among intern nursing students.
- Investigate the direction of correlation between empathy, emotional support, and caring behavior among intern nursing students.
- Assess whether empathy mediates the association between emotional support and caring behavior in intern nursing students.

### Study questions

- How Does Empathy Influence the Relationship Between Emotional Support and Caring Behavior among Intern Nursing Students?
- Investigate the degree to which empathy enhances or mediates the connection between emotional support from nursing students and the subsequent demonstration of caring behavior towards patients.
- What factors impact the caring behavior of intern nursing students?

## Methodology and materials

### Study design

The present study employed a descriptive correlational design.

### Setting &participants

The research was conducted at Alexandria and Damanhur University’s College of Nursing. A total of 450 and 400 nursing students were in their internship years of 2022–2023 at Alexandria and Damanhur University, respectively.

The G*Power Windows 3.1.9.7 software is employed for determining the sample size required for a study, explicitly using calculations based on F tests in the context of linear multiple regression with a fixed model and the R² deviation from zero. The analysis is a priori, aiming to compute the necessary sample size. In this case, the input parameters include an effect size of f² = 0.15, an alpha error probability (α) of 0.05, and a desired statistical power (1-β error probability) of 0.95. The analysis involves 10 predictors. The output from the program provides the noncentrality parameter (λ) as 25.8000000, the critical F value as 1.8899310, numerator degrees of freedom as 10, denominator degrees of freedom as 161, and the total sample size required for the study is calculated to be 195. The initial sample size was calculated as 206 intern nurses, but to account for potential attrition, the researchers opted to recruit a slightly larger sample, targeting 206 students.

The eligibility criteria for this study required participants to be novice intern nurses commencing their duties in September 2022, shaving completed three months of patient care as empathy depends on many factors, including the length of contact. Also, intern students should display consistent attendance and be willing to engage in research activities. Additionally, exclusion criteria were students with psychological illness, irregular attendance, and those over 30 years old to maintain a more homogeneous age group within the sample. This decision helps control for potential variations in life experiences and personal responsibilities while minimizing potential influences from diverse academic experiences or prior professional roles that may differ between younger and older nursing students.

The sample selection process utilized a blind approach, incorporating a systematic randomized technique to identify participants from the overall pool of intern nurses. The steps involved were as follows:

- A comprehensive list containing all nursing interns’ names and relevant data was compiled and digitized into a computer-generated randomization list program (450, 400 for Alexandria, and Damnhour).
- This randomization program selected 206 intern students from this list through a random sampling to ensure fairness and minimize bias.
- Each randomly selected student was screened to identify those meeting the predetermined inclusion criteria and contacted to assess their willingness to participate.
- Six nursing students declined to participate and didn’t match the inclusion criteria, leaving 200 intern students as the final sample size.
- Participants were assigned to the Alexandria or Damanhur group (200), with an equal distribution of 100 students each for Alexandria and Damanhur University (Insert Figure: 1).

The data collection phase spanned two months, from the beginning of July 2023 to the end of August 2023, adhering to stringent guidelines to ensure the validity and reliability of the findings.

### Flow graph

**Fig. 1:**
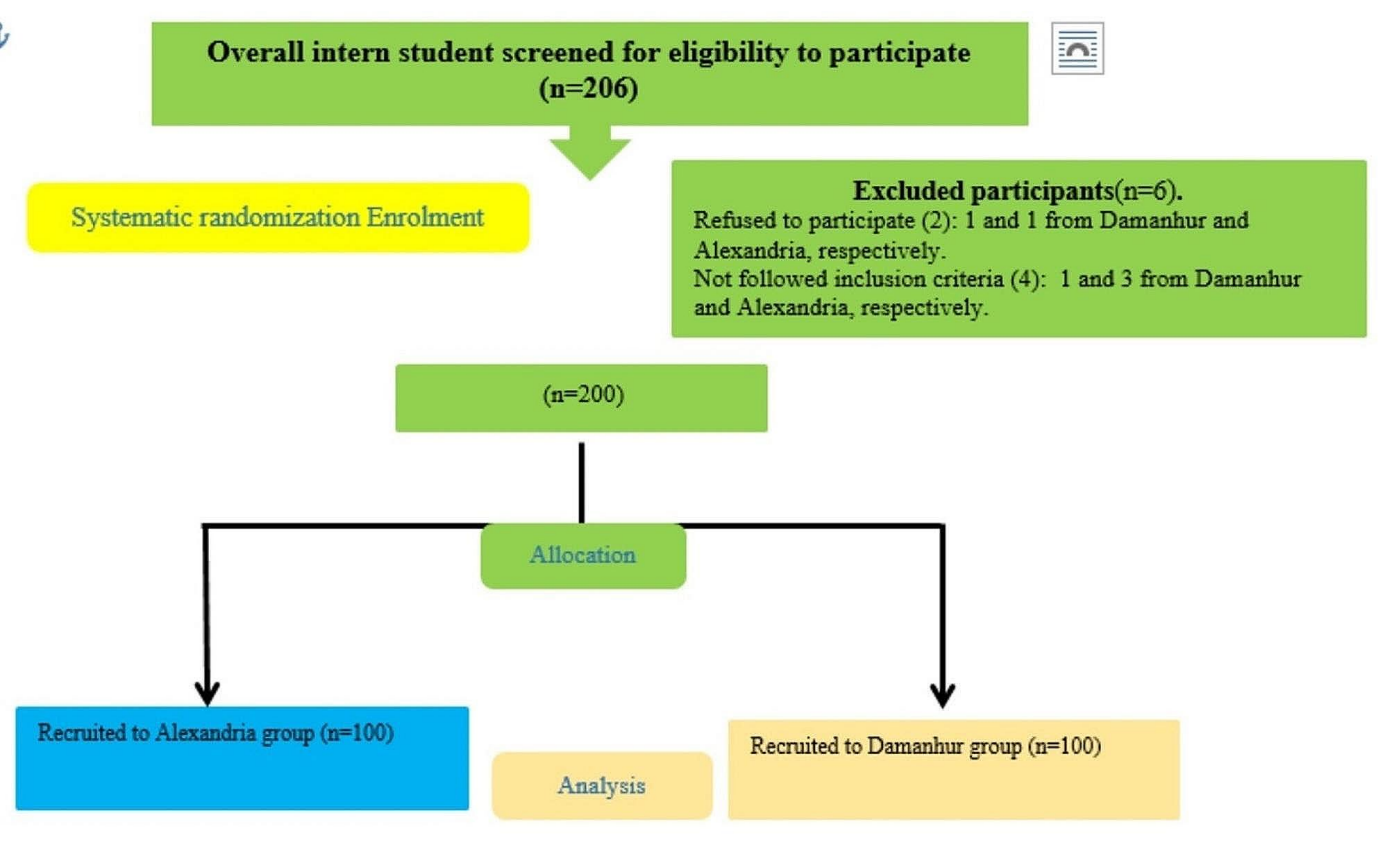
Sample graph

### Measurements

#### This research utilized four distinct tools for data collection

**Tool I: The academic and social form survey** comprised two sections. The first part gathered social information about the students, such as sex, age, place of residency, marital status, monthly expenses, and whether they had a job while studying. The other part gathered academic data, including the overall cumulative GPA and information about extracurricular activities.

**Tool II: The Toronto Empathy Questionnaire (TEQ)** by Spreng et al. (2009) for psychological assessment of an individual’s level of empathy. This scale captures cognitive empathy (the ability to understand others’ emotions) and affective empathy (the ability to share and experience others’ feelings). It consists of 16 items with 8 statements (2, 4, 7, 10, 11, 12, 14, and 15) from the scale that are negatively stated and reversely scored. The responses to statements are rated on a five-point Likert scale that ranges from 0 (never) to 4 (always), with higher scores indicating greater levels of empathy [29]. Xu et al. (2020) found this tool valid and reliable among medical students [30]. Using the Pearson correlation coefficient in the current study, this tool proved to be trustworthy by test-retest (0.74).

**Tool III: Caring Dimension Inventory (CDI)**, developed by Watson & Lea (1997), consists of 25 items to measure different aspects of nursing caring behavior. Patient Responses were rated on a 5-point Likert scale, with higher scores indicating higher caring behavior [31]. The Caring Dimension Inventory is widely used among nursing students and has proven consistent with high reliability [32, 33]. CDI was tested in the current study by test-retest and proved reliable (Pearson correlation coefficient = 0.8).

**Tool IV: The Multidimensional Scale of Perceived Social Support (MSPSS)** is a widely used self-report questionnaire designed to assess how individuals perceive support in various aspects of their lives [34]. The MSPSS consists of 12 items that cover three subscales (family support, friend support, and significant other support), with each item rated on a 7-point Likert-type scale, ranging from 1 (very strongly disagree) to 7 (very strongly agree). Higher scores indicate higher levels of perceived social support from that source. The MSPSS has demonstrated good reliability and validity among nursing students [35]. In the present study, **MSPSS** proved valid and reliable by test-retest with a Pearson correlation coefficient 0.72.

#### Procedures

The research received approval from the Alexandria Research Ethics Committee (**IRB:13,620**). The study obtained further official authorization from the Vice Dean of Students’ Affairs at the Faculty of Nursing in Alexandria and Damanhur, Egypt, granting permission to conduct the research. The contact information of the nursing interns, including their academic email addresses and phone numbers, was obtained from the Internship Affairs unit of Alexandria and Damanhur University and kept confidential.

The researchers developed a tool following a thorough review of the existing literature. A panel of five experts assessed the TEQ, CDI, and MSPSS instruments for face validity and confirmed their validity. Tools II, III, and IV were assessed by conducting a test-retest reliability analysis on a subset of 20 nursing intern students. The results demonstrated high reliability for these instruments.

Before nursing students’ involvement in the study, all participants provided informed consent, with full knowledge that their participation was voluntary and they had the right to withdraw without facing any consequences. A pilot study evaluated the clarity, comprehensibility, and feasibility of the research tools. The findings indicated that the instruments were clear, understandable, and practical for the primary research study. It is important to note that the test-retest reliability assessment involving the intern nursing students and the pilot study were not included in the primary research analysis.

Each recruited subject was interviewed individually to establish rapport and apply tool I, followed by II, III, and IV. Twenty to thirty minutes were needed to complete the data. The collected data were inputted into a computer and analyzed using the IBM SPSS software package version 23.0.

### VII. Statistical analysis

The normality of variable distributions was assessed using the Kolmogorov-Smirnov test. Group comparisons were conducted using the Chi-square test for categorical variables. When comparing more than two categories, the one-way ANOVA test was utilized. The Student t-test was employed to compare two categories of quantitative variables that followed a normal distribution. The Pearson coefficient was used to examine the correlation between customarily distributed quantitative variables. Linear regression was assessed to detect factors that affect the Caring Dimension Inventory. Path analysis was evaluated using AMOS 23. 0 software to detect H’s direct and indirect effects. The Multidimensional Scale of Perceived Social Support on the Caring Dimension Inventory with the Toronto Empathy Questionnaire as a mediator.

## Results

### Socio-demographic characteristics and relations with study variables

Table 1 presents participants’ social and academic data and differences in mean regarding empathy, caring behavior, and emotional support. It was observed that more than half of the participants were males. Regarding their age, approximately two-thirds of the participants fell within the age range of 23 to less than 25 years, with total Mean (SD) = 23.76 (3.6).

Table 1 also indicates a statistically significant relation between sex, age, residence, marital status, monthly expense, and private work and the mean of empathy (*p* < 0.001, *p* = 0.004, *p* < 0.001, *p* = 0.035, *p* = 0.025, and *p* < 0.001, respectively). Regarding caring behavior, a statistically significant relationship was observed with sex, age, monthly expense, and private work (*p* < 0.001, *p* = 0.049, *p* = 0.013, and *p* = 0.005, respectively). Finally, a statistically significant relationship was observed between sex, age, monthly expense, and private work about the mean of emotional support (*p* < 0.001, *p* = 0.003, *p* = 0.001, *p* < 0.001, & *p* < 0.001, respectively).

**Table 1:** Participants’ socio-demographic characteristics and differences in mean and SD regarding empathy, caring behavior, and emotional support

Socio-demographic characteristics (*n* = 200)		No.	%	Empathy (TEQ)	Caring behaviour (CDI)	Emotional support (MSPSS)
**Sex**	Male	106	53.0%	84.92 ± 18.90	33.50 ± 4.85	48.69 ± 15.32
	Female	94	47.0%	100.10 ± 11.61	41.48 ± 8.63	62.80 ± 14.73
	**t(p)**			**6.925** ^*^ **(< 0.001** ^*^ **)**	**7.923** ^*^ **(< 0.001** ^*^ **)**	**6.619** ^*^ **(< 0.001** ^*^ **)**
**Age (years)** **Mean (SD) = 23.76 (3.6)**	19 > 21	4	2.0%	85.75 ± 10.47	41.75 ± 12.26	56.00 ± 17.51
	21 > 23	36	18.0%	101.03 ± 10.09	34.56 ± 6.98	64.00 ± 13.72
	23 > 25	135	67.5%	90.94 ± 18.50	38.10 ± 8.14	54.10 ± 16.46
	≥ 25	25	12.5%	86.12 ± 17.91	35.80 ± 6.61	49.28 ± 17.18
	**F(p)**			**4.670** ^*^ **(0.004** ^*^ **)**	**2.669** ^*^ **(0.049** ^*^ **)**	**4.909** ^*^ **(0.003** ^*^ **)**
**Residence**	Urban	135	67.5%	88.63 ± 18.86	36.79 ± 7.61	52.64 ± 15.77
	Rural	65	32.5%	99.15 ± 11.88	38.22 ± 8.59	60.88 ± 16.98
	**t(p)**			**4.800** ^*^ **(< 0.001** ^*^ **)**	**1.144(0.255)**	**3.372** ^*^ **(0.001)** ^*^
**Marital stats**	Single	153	76.5%	90.86 ± 18.72	37.35 ± 8.05	54.93 ± 16.59
	Married	47	23.5%	95.94 ± 12.63	36.94 ± 7.68	56.57 ± 16.70
	**t(p)**			**2.130** ^*^ **(0.035** ^*^ **)**	**0.309(0.758)**	**0.592(0.555)**
**Monthly expense** Average income in dollar, mean (SD): 263.80 (95.99)	inadequate	97	48.5%	89.15 ± 20.42	35.82 ± 7.35	52.06 ± 16.41
	Adequate	103	51.5%	94.78 ± 13.98	38.59 ± 8.28	58.39 ± 16.24
	**t(p)**			**2.259** ^*^ **(0.025** ^*^ **)**	**2.502** ^*^ **(0.013** ^*^ **)**	**2.740** ^*^ **(0.007** ^*^ **)**
**Private Work**	No	65	32.5%	99.49 ± 11.63	39.60 ± 8.24	59.51 ± 16.11
	Yes	135	67.5%	88.47 ± 18.84	36.12 ± 7.57	53.30 ± 16.49
	**t(p)**			**5.080** ^*^ **(< 0.001** ^*^ **)**	**2.871** ^*^ **(0.005)** ^*^	**2.510** ^*^ **(0.013** ^*^ **)**

F: One-way ANOVA test t: Student t-test *: Statistically significant at *p* ≤ 0.05

Table 2 shows participants’ academic characteristics and differences in mean regarding empathy, caring behavior, and emotional support. It was observed that intern nursing students affiliated with Alexandria University had a higher mean of empathy, caring behavior, and emotional support than those affiliated with Damanhur University. Participants’ affiliation revealed significant variations in caring behavior and emotional support (*p* < 0.001), while an insignificant relationship was noted between empathy and participants’ affiliation (*p* = 0.171). The results show that students who didn’t participate in social or academic activities had the highest mean of empathy, caring behavior, and emotional support, with a statistically significant difference (*p* = 0.001, *p* = 0.021& *p* < 0.001respectively. The table also illustrates that the studied students who obtained excellent or very good grades had the highest mean of empathy, caring behavior, and emotional support, with a statistically significant difference (*p* < 0.001).

**Table 2:** Participants’ academic characteristics and differences in mean regarding empathy, caring behavior, and emotional support

Academic characteristics (*n* = 200)		No.	%	Empathy (TEQ)	Caring behaviour (CDI)	Emotional support (MSPSS)
**University**	Alexandria	100	50%	38.0 ± 8.5	98.6 ± 12.2	60.4 ± 12.5
	Damnhour	100	50%	36.5 ± 7.3	85.5 ± 19.6	50.3 ± 18.6
	**t(p)**			1.373 **(**0.171**)**	5.674 ^*^(< 0.001^*^)	4.530^*^(< 0.001^*^)
**Extra Curricula activities**	No	139	69.5%	94.94 ± 15.86	38.06 ± 8.17	58.53 ± 16.07
	Yes	61	30.5%	85.46 ± 19.58	35.39 ± 7.14	48.02 ± 15.52
	**t(p)**			**3.333** ^*^ **(0.001** ^*^ **)**	**2.330** ^*^ **(0.021** ^*^ **)**	**4.303** ^*^ **(< 0.001** ^*^ **)**
**Total GPA**	Fair	3	1.5%	93.33 ± 4.16	36.00 ± 11.27	60.67 ± 11.02
	Good	74	37.0%	86.15 ± 18.82	33.84 ± 5.41	50.41 ± 15.60
	Very good	100	50.0%	93.45 ± 16.66	38.12 ± 7.95	56.17 ± 16.48
	Excellent	23	11.5%	104.78 ± 8.64	44.61 ± 8.89	66.74 ± 14.89
	**F(p)**			**7.724** ^*^ **(< 0.001** ^*^ **)**	**13.768** ^*^ **(< 0.001** ^*^ **)**	**6.487** ^*^ **(< 0.001** ^*^ **)**

F: One-way ANOVA test t: Student t-test *: Statistically significant at *p* ≤ 0.05

### Study measures and correlations

Table 3 reveals the association between empathy, caring behavior, and emotional support levels among the studied students. It can be noticed that a significant positive correlation was found between empathy, caring behavior, and emotional support (*P* < 0.001). This means that a high level of empathy indicates a higher level of caring behavior and emotional support.

**Table 3:** Correlation between empathy, caring behavior, and perceived social support among studied students

Study variables (*n* = 200)	Empathy (TEQ)		Emotional support (MSPSS)	
	*r*	*P*	*r*	*p*
**Caring behavior (CDI)**	0.420^*^	< 0.001^*^	0.779^*^	< 0.001^*^
**Emotional support (MSPSS)**	0.467^*^	< 0.001^*^		

r: Pearson coefficient *: Statistically significant at *p* ≤ 0.05

**Fig. 2:**
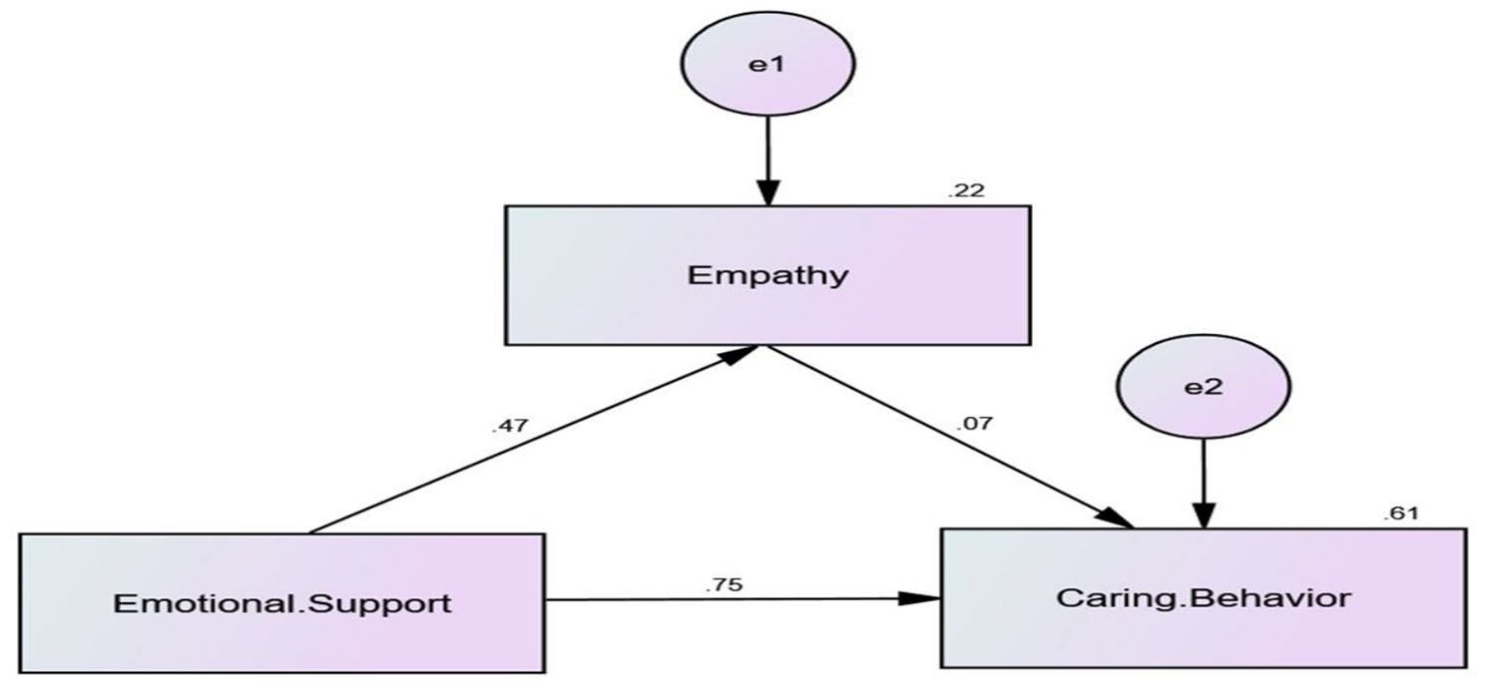
The path analysis of the standardized coefficients assessed emotional support’s direct and indirect effects on caring behavior, with empathy mediating

Figure 2; Table 4 present the results of the standardized regression weights, standard error (SE), critical ratio (CR), and significance (p-value) for both the direct and indirect effects of emotional support on caring behavior mediated by empathy. These results were generated using SPSS-AMOS.

### Regression analysis: predictors of caring behavior

Table 4 delineates the findings of a path analysis scrutinizing the direct and indirect effects of emotional support on caring behavior within the domain of intern nursing students, with empathy as a mediator. Significantly, a positive and statistically significant direct impact of emotional support on empathy is evident, underscoring the association between increased emotional support and heightened levels of empathy among intern nursing students. Moreover, a substantial direct effect of emotional support on caring behavior is highlighted, suggesting a strong link between augmented emotional support and intensified caring behavior towards patients during the internship.

However, while present, the indirect effect of empathy on caring behavior is quantitatively limited and lacks statistical significance at the conventional level. The modest impact of empathy in mediating the relationship between emotional support and caring behavior suggests that other factors may contribute more substantially to the observed caring behavior among intern nursing students.

The model demonstrates a commendable fit to the data, supported by Comparative Fit Index (CFI) and Incremental Fit Index (IFI) values close to 1.000 and a reasonable Root Mean Square Error of Approximation (RMSEA).

**Table 4:** A path analysis of direct and indirect effects of emotional support on caring behavior mediated by empathy

Variable 1		Variable 2	Standardized regression weights (Direct effect)	S.E	C.*R*	*p*-value	Indirect effect
Empathy	←	**Emotional Support**	0.224	0.030	7.445^*^	< 0.001^*^	0.0
Caring. Behavior	←	**Emotional Support**	0.791^*^	0.053	14.930^*^	< 0.001^*^	0.035
Caring. Behavior	←	**Empathy**	0.159	0.111	1.435	0.151	0.0

Model fit parameters CFI; IFI; RMSEA (1.000; 1.000; 0.060)

CFI stands for comparative fit index, IFI for incremental fit index, and RMSEA for Root Mean Square Error of Approximation

Model ; significance 79.008^*^(< 0.001^*^)

Table 5 presents factors predicting caring behavior among the studied subjects using multiple linear regression analysis. It can be noticed that caring behavior significantly increased with the increased intern nursing students’ emotional support and among those who were not working in private hospitals (*p* < 0.001,&*p* = 0.023 respectively) with a regression coefficient of 0.644. Other sociodemographic characteristics and empathy were not significant predictors of caring behavior in the model.

**Table 5:** Factors predicting caring behavior among studied subjects using multiple linear regression analysis

Study Variables	B	Beta	t	*p*	95% CI	
					LL	UL
Sex (Female)	1.057	0.030	0.508	0.612	-3.045	5.160
Age (Old age)	0.041	0.003	0.061	0.952	-1.301	1.384
Residence (Rural)	2.333	0.062	1.241	0.216	-1.374	6.040
Marital status (Married)	0.864	0.021	0.405	0.686	-3.340	5.067
Income (Enough)	-0.086	-0.002	-0.053	0.958	-3.284	3.111
Work (Yes)	-4.485	-0.120	**-2.300** ^*^	**0.023** ^*^	-8.332	-0.639
Extra Curricula Activities (Yes)	0.967	0.025	0.536	0.592	-2.589	4.523
GPA (Very good/ Excellent)	1.482	0.058	1.143	0.254	-1.075	4.038
Empathy (TEQ)	0.054	0.025	0.435	0.664	-0.193	0.301
Emotional support (MSPSS)	0.755	0.712	**13.100** ^*^	**< 0.001** ^*^	0.641	0.869

R^2^ = 0.644,F = 34.164^*^,*p* < 0.001^*^

F, p: f and p values for the model

R^2^: Coefficient of determination

B: Unstandardized Coefficients

Beta: Standardized Coefficients

t: t-test of significance

LL: Lower limit UL: Upper Limit

*: Statistically significant at *p* ≤ 0.05

## Discussion

As nursing students progress through their training, they encounter challenging and emotionally charged situations, such as witnessing patients’ suffering and dealing with family and patients’ grief and loss. During these moments, the value of receiving emotional support from peers, family, mentors, and faculty members becomes immense. This support creates safe and empathetic opportunities for nursing students to process their emotions, manage stress, and preserve their well-being. Consequently, it cultivates resilience, compassion, and empathy, which help them develop stronger connections with patients [36].

Intern nursing students receiving emotional support tend to exhibit caring behaviors, such as active listening, comforting touch, effective communication, and personalized care. They comprehend the significance of addressing not just the physical needs of patients but also their emotional and psychological well-being. Integrating emotional support into their practice prepares nursing students to deliver holistic care, fostering a therapeutic environment for healing and recovery [37, 38]. Therefore, this study explored the association between empathy, emotional support, and caring behavior among intern nursing students.

The study findings revealed a significant positive correlation between empathy and caring behavior. This may be because empathy is one dimension of caring behavior, so intern nursing students with higher levels of empathy are more likely to exhibit more remarkable care development, leading to increased accountability, ethical problem-solving, and overall growth in providing care. These findings align with previous studies emphasizing the link between nursing students’ caring behaviors and empathy [39–41].

The study also found a significant association between emotional support and caring behavior. This result is supported by linear regression analysis in the present study, which showed that emotional support is one predictor of caring behavior toward the patient. This suggests that providing emotional support from families, peers, or others creates a nurturing and supportive environment that fosters a sense of concern, understanding, and appreciation among nursing students. Therefore, they can address patients’ emotional needs, utilizing active listening, offering reassurance, establishing meaningful connections, promoting trust, and delivering patient-centered care. This, in turn, enhances their caring behavior towards patients [42].

Moreover, the present study’s results revealed a statistically significant correlation between emotional support and empathy among intern nursing students. This suggests that individuals receiving higher emotional support tend to empathize more with their patients. Empathetic individuals are more attuned to others’ emotional states, better able to recognize when emotional support is needed, and more likely to respond in a caring and compassionate manner, alleviating distress and promoting well-being. Empathy creates a sense of connectedness, fostering trust and openness in relationships (27.42).

Furthermore, empathy is crucial in the relationship between emotional support and caring behavior toward patients among intern nursing students. Emotional support received by nursing students can profoundly impact their empathetic abilities. When students feel supported and understood by their educators, family members, and peers, they are more likely to develop a heightened empathy toward patients. Korkmaz Doğdu et al. (2022) concluded that increasing empathy among nursing students catalyzes demonstrating caring behaviors during patient interactions [18]. The mediating role of empathy is significant because it bridges the gap between the emotional support received by nursing students and their subsequent caring behavior towards patients. It has been proven that health professionals with high levels of empathy operate more efficiently to fulfill their role in eliciting therapeutic change [27]. .

Concerning the current nursing students’ social data and their relation to empathy, the present study found that younger nursing interns demonstrated significantly higher mean empathy than older nursing interns. This may be attributed to the influence of social environments, social prejudices, stereotypes, and racial bias, which can reduce empathic responses [43, 44]. Regarding sex differences, female nursing students exhibited a higher mean of empathy compared to their male peers. This finding is consistent with previous studies attributing higher female empathy levels to their perceived emotional sensitivity and societal gender role norms [42–44]. Additionally, marital status impacted empathy, with married nursing students demonstrating the highest mean score. This may be related to the role of mothers in providing care, love, and support to their spouses and children. According to the author, the conflicting results could be due to the low percentage of married students (25.2%) in this study, which has resulted in biased results. This result aligns with a previous study that reported married nursing interns scored higher in the empathic concern subscale than unmarried nursing interns. This means that married nursing interns felt more sympathy and caring for others than unmarried nursing interns [1].

The study examined the academic characteristics of the studied students and their relation to empathy. It revealed a statistically significant positive association between empathy and the grade point average (GPA) of intern nursing students. This suggests that empathy is a skill that can be cultivated through educational processes, and higher-achieving students tend to exhibit higher levels of empathy [45–48]. However, some studies have reported no significant changes in empathy levels across different GPA points among nursing interns [49–51].

The study also examined the mean of empathy, emotional support, and caring behavior among intern nursing students related to University affiliation. Nursing students affiliated with Alexandria University had a higher mean compared to those affiliated with Damanhur University. These differences may be attributed to variations in the development of these competencies within each nursing program. The absence of a unified nursing curriculum between the two Universities highlights the need for a national unified nursing curriculum and standardized nursing education to ensure the consistent development of empathy and caring behavior among nursing interns (51,52).

In conclusion, emotional support is a crucial attribute for intern nursing students. This support helps them alleviate stress, boost their self-confidence, and contribute to their overall well-being. As a result, they are more likely to demonstrate caring behavior towards their patients and colleagues, resulting in improved patient outcomes and satisfaction.

### Implications

The study underscores the considerable importance of emotional support received from others and empathy in promoting caring behavior among intern nursing students. Our findings emphasize the critical need for empathy training within nursing curricula. By investing in programs that enhance students’ ability to empathize with patients, educators can nurture a more compassionate approach to care. Additionally, our results highlight the importance of providing emotional support to nursing students, particularly during internships. Creating a supportive environment where students feel valued can foster empathy development and ultimately enhance patient care. Moreover, the study underscores the significance of tailoring support for interns, especially those working in non-private hospitals. Implementing strategies to assist interns in navigating challenges and promoting a culture of support can facilitate the cultivation of caring behaviors. Lastly, understanding the mediating role of empathy sheds light on the interconnectedness of empathy, emotional support, and caring behavior. This insight can inform the design of interventions to strengthen empathy as a pathway to improving patient outcomes. Our study underscores the vital role of empathy and emotional support in nursing education and practice, urging healthcare institutions to prioritize these aspects to cultivate a workforce of compassionate and empathetic nurses.

.

### Limitations

The study focused on intern nursing students, which limits the generalization of the findings to other healthcare professionals or individuals in different stages of their nursing education. Further research with a more diverse sample is needed to validate the findings in various contexts. The research relies on self-reported data, which may be subject to social desirability bias or inaccuracies due to subjective interpretations. Future studies could incorporate additional objective measures or observational data to strengthen the validity of the findings.

## Conclusion

Our study sheds light on the intricate dynamics between empathy, emotional support, and caring behavior among intern nursing students. The findings underscore the significant association between empathy, caring behavior, and emotional support, highlighting the pivotal role of empathy in fostering compassionate care. Significantly, higher levels of empathy were correlated with increased levels of caring behavior and emotional support, emphasizing the importance of nurturing empathy skills within nursing education and practice.

Furthermore, our results reveal the nuanced influence of emotional support on caring behavior, particularly among intern nursing students. The study demonstrates that providing emotional support is associated with enhanced caring behavior, especially in environments outside private hospitals. This underscores the need for tailored support systems to assist interns, particularly in non-private hospital settings, to foster a culture of empathy and compassion.

Moreover, our findings indicate that empathy is a mediating factor in the relationship between emotional support and caring behavior. This suggests that interventions promoting empathy may serve as a pathway to enhancing caring behavior among nursing students. Understanding the mediating role of empathy offers valuable insights into strategies for improving patient care outcomes by strengthening empathy skills among healthcare professionals.

## Data Availability

No, I don’t have any research data outside the submitted manuscript file.
